# TAK1 inhibition prevents the development of autoimmune diabetes in NOD mice

**DOI:** 10.1038/srep14593

**Published:** 2015-10-13

**Authors:** Hui Cao, Jingli Lu, Jiao Du, Fei Xia, Shouguo Wei, Xiulan Liu, Tingting Liu, Yang Liu, Ming Xiang

**Affiliations:** 1 Department of Pharmacology, School of Pharmacy, Tongji Medical College, Huazhong University of Science and Technology, Wuhan, China; 2 Synergy Innovation Center of Biological Peptide Antidiabetics of Hubei Province, School of Life Science, Wuchang University of Technology, Wuhan, China

## Abstract

Transforming growth factor-β activated kinase-1 (TAK1, Map3k7), a member of the mitogen-activated protein kinase kinase kinase (MAP3K) family, is essential in innate and adaptive immune responses. We postulated that blockade of TAK1 would affect autoimmune diabetes in non-obese diabetic (NOD) mice. Administration of 5Z-7-oxozeaenol (OZ), a TAK1 inhibitor, decreased the incidence and delayed the onset of autoimmune diabetes in both spontaneous and accelerated (cyclophosphamide-induced) experimental NOD mice. OZ also reduced insulitis, preserved islet function, increased the expression of α1- antitrypsin (AAT), and severely inhibited NF-κB and JNK/AP-1 signaling pathways in immune organs and pancreatic tissues. Importantly, TAK1 inhibition by OZ elicited a Th1 to Th2 cytokine shift, and increased TGF-β1 production in cultured T lymphocytes supernatants. Systemic TAK1 inhibition induced immature DCs with lower expressions of MHC-II and CD86, attenuated DC-mediated T cell proliferation in allogeneic MLR, and production of cytokine IL-12p70 in DCs suspensions. The results indicate that TAK1 inhibition with OZ was associated with a lower frequency of autoimmune diabetes in NOD mice. The net effect of TAK1 inhibition in NOD mice therefore appears to be protective rather than disease-enhancing. Strategies targeting TAK1 specifically in NOD mice might prove useful for the treatment of autoimmune diabetes in general.

Type 1 diabetes mellitus (T1DM) is a chronic autoimmune disorder caused by autoreactive T cells, which mediates the impairment of insulin-producing pancreatic β-cell function^1^,^2^. Insulin replacement is the mainstay of treatment for T1DM, but its disadvantages include poor effectiveness in preventing long-term complications, frequency of episodes of severe hypoglycemia, and disruption of lifestyle^3^,^4^. In addition, insulin treatment does not inhibit T cell-mediated β cell function^5^,^6^. Strategies aimed at stopping immune destruction of β cells and preserving β cell function may thus improve overall T1DM therapy.

The NOD mouse is a spontaneous model of type 1 diabetes, with genetic and pathophysiological characteristics that are similar to those of the human disease^7^. Early islet inflammation probably involves T-cell infiltration at the endocrine/exocrine border. Consequent “peri-insulitis” lasts for weeks to months in NOD mice and likely for years in humans before detectable β-cell death^8^. During pre-insulitis, islet antigen is initially presented by dendritic cells (DCs) to islet antigen-specific T cells and innate immunity occurs^9^. As ‘danger signals’ such as cytokines and chemokines are released by dying β cells and immune cells, immune cells are activated and attracted to pancreatic islets (a process termed insulitis) to destroy β cells^10^. These immune cells include T cells, B cells, macrophages, natural killer (NK) cells and NKT cells, as well as DC subsets contributing to β cells death^11^. Thus, development of T1DM involves complex interactions between immune cells and pancreatic β cells.

TAK1 (transforming growth factor-β-activated kinase-1, Map3k7), a member of the mitogen-activated protein kinase kinase kinase (MAP3K) family, functions as a critical regulator in innate and adaptive immune responses^12^,^13^,^14^. Many of the signaling pathways triggered by multiple extracellular stimuli converge at the level of TAK1. Those stimuli include cytokines such as interleukin-1 (IL-1), toll-like receptor (TLR), tumor necrosis factor (TNF), transforming growth factor β (TGF-β), B cell receptor (BCR), and T cell receptor (TCR) ligands^15^,^16^. Activated TAK1 then phosphorylates the IKK complex as well as p38, c-Jun N-terminal kinase (JNK), and extracellular signal regulated kinase (ERK), thus activating NF-κB and AP-1 17. Ultimately, these transcription factors initiate expression of genes involved in inflammatory responses. Subsequent IKK activation induces the expression of cytokines, chemokines, and adhesion molecules that mediate the recruitment and activation of immune cells^18^. In addition, TAK1 induces expression of antiapoptotic proteins to protect cells from cytokine-induced death^19^. Therefore, as a key regulator of downstream signaling pathways, TAK1 is implicated in a number of pathophysiologic processes including CNS autoimmune inflammation, arthritis, and colitis^20^,^21^,^22^.

TAK1 conditional knockout systems have been used to reveal roles of TAK1 in immune cells including T cells, B cells, DCs, and Gr-1^+^CD11b^+^ neutrophils. In B cells and T cells, TAK1 is required for development and survival through NF-κB and MAPK pathways induced by cytokines, TLR ligands, and T cell receptor (TCR)-or B cell receptor (BCR)^12^,^23^,^24^. In DCs, TAK1 acts to maintain mature DCs and BM precursors^25^. DC cell-specific ablation of TAK1 causes a myeloid proliferative disorder, disrupts T-cell homeostasis, and prevents effective T-cell priming and Tregs generation^25^. However, TAK1 may also negatively regulate TLR4-induced NF-κB and p38 signaling pathways during myeloid cell homeostasis^26^. The role of TAK1 in controlling the immune system *in vivo*, and autoimmune diabetes in particular, is complex and not yet fully understood. Here, we investigated the role of TAK1-dependent cascades in a preclinical mouse model of autoimmune diabetes using the TAK1 inhibitor 5Z-7-oxozeaenol (OZ). The results provide evidence that TAK1 may be a potential target for treatment of T1DM.

## Results

### TAK1 inhibition affects maturation and survival of DCs via interfering with NF-κB and JNK/AP-1 signaling pathway

**Figure 1:**
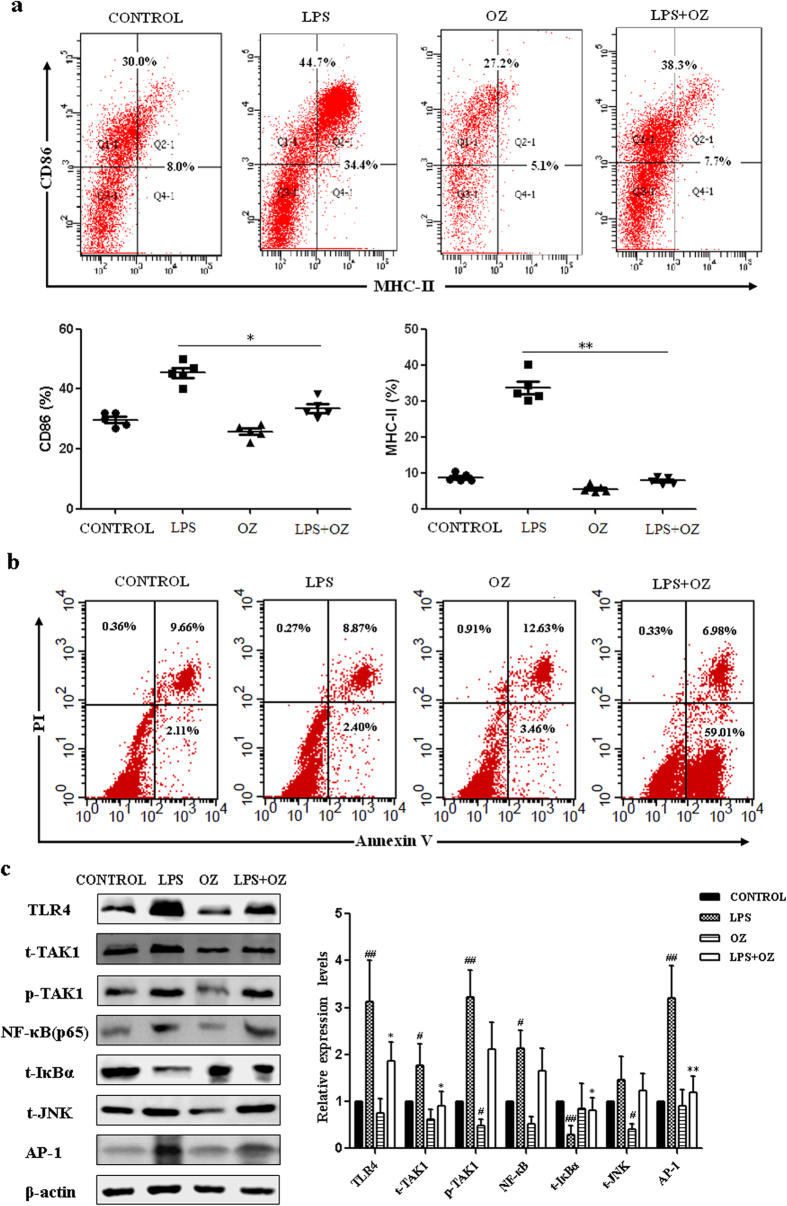
Effect of TAK1 inhibition in DCs. (**a**) Effect of TAK1 inhibition on DCs’ development. OZ-treated DCs with LPS stimulation expressed low CD86 and MHC-II, indicating TAK1 affected DCs maturation. (**b**) Effect of TAK1 inhibition on apoptosis in mature DCs. The apoptosis of mature DCs were analyzed by flow cytometry. TAK1 inhibitor promoted mature DCs’ apoptosis. (**c**) Effect of TAK1 inhibition on LPS-induced NF-κB and JNK/AP-1 signaling in DCs. DCs were generated from bone marrow of C57BL/6 mice, the cells were treated with 1 μg/ml LPS for 24 h, then cells were treated with either DMSO vehicle or 5 μM OZ for 4 h. Blockade of TAK1 leads to a reduction of LPS-induced NF-κB and JNK/AP-1 signaling as a downstream indicator of TAK1 signaling activity. Representative data from three or four independent experiments were expressed as mean ± SD. β-actin was detected as the internal reference. ^#^P < 0.05, compared with control DCs; *P < 0.05, **P < 0.01, compared with LPS-treated DCs.

To determine the role of TAK1 in differentiation and maturation of DCs, we examined the development of DCs derived from bone marrow of C57BL/6 mice. We firstly assessed OZ cytotoxicity in DCs. OZ showed minimal cytotoxicity with more than 90% cell viability at the concentration of 20 μM (Supplementary Fig. S1a online). Cells in the bone marrow were treated with either DMSO vehicle or 5 μM OZ for 4 h.The number of total CD11c^+^DCs were comparable between OZ-treated DCs and control DCs, indicating that TAK1 inhibitor did not affect the differentiation of bone marrow cells into DCs (Fig. 1a). Among the populations of CD11c-gated cells, OZ-treated DCs (with LPS stimulation) expressed lower CD86 and MHC-II than LPS-stimulated DCs did, demonstrating TAK1 inhibited DCs maturation (Fig. 1a).

To further study the effect of TAK1 inhibition on DCs, we measured the extent of apoptosis in DCs which were treated with 5 μM OZ. Apoptosis was assayed by FITC annexin V/PI staining in combination with FACS analysis. Mature DCs were derived from bone marrow and then stimulated with GM-CSF plus IL-4 and LPS. The results showed that OZ treatment produced significant apoptosis in mature but not in immature DCs (Fig. 1b). Approximately 50% of OZ-treated mature cells were apoptotic.

To determine the effects of TAK1 on the activity of TLR4-induced signal transduction in DCs, LPS was used to activate the TLR4 pathway^27^. Bone marrow-derived DCs were treated with 5 μM of the TAK1 inhibitor, 1 μg/mL of LPS, or both. Compared with the control group, OZ-treated DCs significantly diminished the expression of TLR4 and NF-κB p65 both in protein and mRNA level, indicating that TAK1 influenced the survival of DCs. We also detected the activity of JNK/AP-1 signaling pathway in OZ-treated DCs. TAK1 inhibition led to a reduction of JNK and AP-1 activation in DCs. (Fig. 1c and Supplementary Fig. S2a online)

### TAK1 inhibitor impacts the percentage of Tregs *in vitro*

Firstly, we tested the cytotoxicity of OZ in T cells. As shown in supplementary Fig. S1b, there were 90% cell viability at the concentration of 20 μM, which indicated that OZ had minimal cytotoxicity directly on T cells. Tregs participate in the regulation of the immune state during the progression of T1D^28^. In this study, OZ decreased the differentiation of CD4^+^CD25^+^Foxp3^+^ Tregs from spleen, thymus and lymph nodes of C57BL/6 mice (Supplementary Fig. S3a–c online). OZ also ameliorated the gene expression of Foxp3, an essential factor for the differentiation and function of Tregs (Supplementary Fig. S3d online).

### TAK1 inhibitor downregulates the mRNA levels of TAK1 and its downstream components *in vitro*

To explore possible mechanisms of TAK1 inhibitor -OZ in immunosuppression *in vitro*, we carried out the following experiments focusing on TAK1 and associated signal pathways. We estimated mRNA expressions of key molecules, includingTLR4, TAK1, NF-κB, IκBα, JNK and AP-1 in T cells from spleen, thymus, and lymph nodes. Results manifested that OZ decreased the gene expressions of TLR4, TAK1, NF-κB, JNK and AP-1, and enhanced IκBα expression (Supplementary Fig. S2b-d online). These data indicated that OZ executed immunoregulatory effects of T cells *in vitro* via inhibiting the activation of NF-κB and JNK/AP-1 signaling pathway.

### TAK1 expression increases during development of insulitis

**Figure 2:**
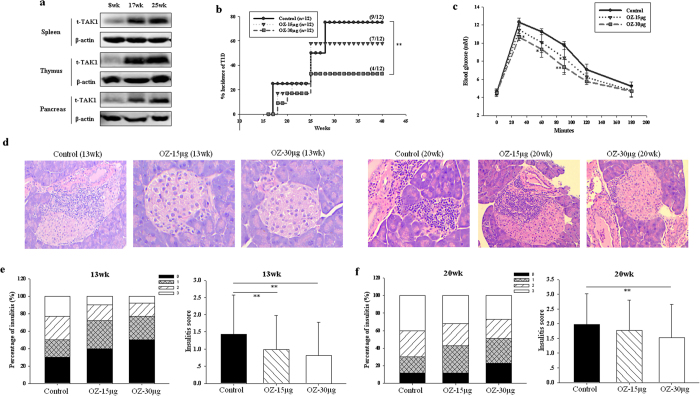
Impact of TAK1 inhibitor on spontaneous diabetes onset in female NOD mice. Spontaneous autoimmune diabetes onset was evaluated in female NOD mice exposed to either 15 μg/mouse/week OZ, 30 μg/mouse/week OZ, or PBS treatment starting at age 8 weeks up to age 11 weeks. (**a**) Expression level of t-TAK1 protein in immune organs and pancreas from 8, 17 or 25-week-old NOD mice. (**b**) TAK1 inhibitor delayed the onset and decreased the incidence of autoimmune diseases in NOD mice. (**c**) Glucose tolerance of non-diabetic NOD mice at age of 12 weeks. Glucose was measured in blood from the tail vein at 0, 30, 60, 90, 120, 180 min after glucose administration. (**d**) Representative images of pancreatic islets from 13 and 20-week-old NOD mice. (**e**,**f**) Distribution of insulitis scores at age of 13 weeks and 20 weeks. Scoring scale: 0, no insulitis; 1, peri-insulitis; 2, <50% invasive islet damage; and 3, >50% islet mass destroyed. Average percentages of insulitis were determined from 50 to 100 islets in each treatment group. Data represent the mean ± SD (n=6 in each group). *P < 0.05, **P < 0.01 for Control vs. OZ (15 μg or 30 μg/mouse)

In our NOD female mice, nondestructive peri-insulitis developed from 6 to 10 weeks of age followed by an invasive insulitis at 15 weeks. The onset of diabetes was first observed at age of 17 weeks, progressing to a cumulative disease incidence of 60–80% by 40 weeks of age. Results of western analysis showed that, compared to 8-week-old mice, TAK1 protein was significantly increased in 17-week-old mice and reached a maximum by 25 weeks of age in thymus, spleen, and pancreas (Fig. 2a). Immunohistochemistry analysis manifested that TAK1 was expressed in the blood vessels of pancreas in the absence of insulitis and increased with disease progression (Supplementary Fig. S4 online). Thus, levels of TAK1 in NOD mice increase in an age-dependent manner, suggesting that TAK-1 is associated with the progression of T1DM.

### TAK1 inhibition reverses T1DM in early-onset NOD mice, but not late-onset NOD mice

To further validate TAK1 as a candidate therapeutic target in the amelioration of diabetes, we used a potent and selective TAK1 kinase inhibitor—5Z-7-oxozeaenol (OZ) to treat female NOD mice at the onset of diabetes with doses of 15 μg or 30 μg/mouse. As shown in Fig. 2b, OZ at 30 μg/mouse normalized hyperglycemia. OZ also delayed or reduced the incidence of diabetes in a dose dependent manner. However, OZ at 5 μg/mouse had no effect on glucose control (data not shown). Administration of OZ (30 μg/mouse) to 8-week-old female NOD mice for four weeks significantly decreased diabetes incidence to 33% in comparison to mice treated with PBS, whose diabetes incidence was 75%. Notably, 8 of 12 OZ-treated mice remained diabetes-free after cessation of treatment, with some remaining in remission up to 40 weeks. These results showed that TAK1 inhibition can have long-term protective effects in NOD mice. As an aside, we also found that OZ treatment reduced glucose levels of nonfasted diabetic mice. We also observed that the effectiveness of TAK-1 inhibition was dependent on viable islet mass in the early stage of diabetes; we found no efficacy of OZ if diabetic mice had no or very few remaining islets prior to treatment (data not shown).

### TAK1 inhibition reduces insulitis and preserves islet function

We next examined whether amelioration of the diabetic state with the TAK1 inhibitor was associated with reduced insulitis and improved islet function in NOD mice. Examination of islet infiltration after drug administration showed that the percentage of severely infiltrated islets (score = 3) in diabetic mice treated with OZ for 4 weeks was significantly reduced compared to islets of untreated mice (Fig. 2d–f). The OZ-treated NOD mice thus had significantly more islets free of mononuclear cell infiltration than did the control mice. Intraperitoneal glucose tolerance test (IPGTT) assays revealed improved glucose tolerance 2 weeks after OZ treatment of prediabetic mice (Fig. 2c), consistent with reduced insulitis and preserved islet function.

**Figure 3:**
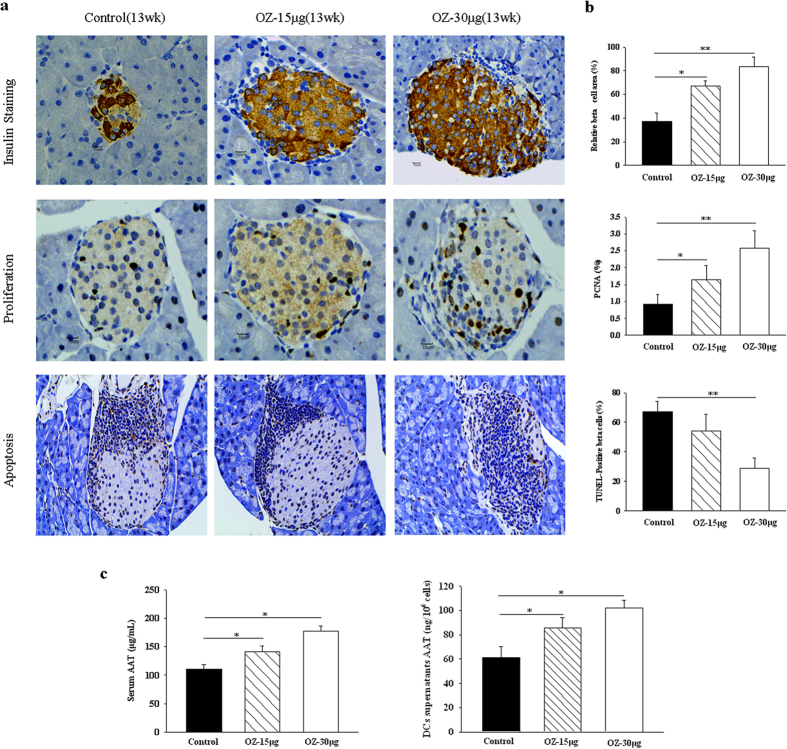
TAK1 inhibition protects β cells. OZ was administered to 8-week-old female NOD mice at doses of 15 μg or 30 μg/mouse once a week for 4 weeks, respectively. Pancreata were removed at age of 13 weeks. (**a**,**b**) Immunostaining for insulin, proliferation and apoptosis on spontaneous diabetes onset in female NOD mice. In the pancreas of OZ-treated NOD mice, islets were larger, insulin-positive cells were more, and apoptotic β cells was significantly lower than those in the PBS-treated mice. (**c**) Effects of TAK1 inhibition on AAT secretion in DCs’ supernatants and serum. Cell supernatants and serum were collected, and AAT levels were measured by ELISA. Data represent the mean ± SD (n = 6 in each group). *P < 0.05, **P < 0.01 for Control vs. OZ (15 μg or 30 μg/mouse)

To determine the extent of apoptosis in β cells, sections were stained for TUNEL, insulin, and PCNA (proliferating cell nuclear antigen). The number of apoptotic cells was higher in NOD mice treated with PBS at 13 weeks of age than in sections from OZ-treated mice (Fig. 3). TUNEL-positive β cells in PBS- and OZ-treated (30 μg/mice) NOD mice were 67.0  ± 6.71% and 29.0 ± 6.46%, respectively. As a result, TAK1 inhibition in NOD mice caused a 48.1% decrease in the relative number of apoptotic β cells. To examine the proliferation of pre-existing β cells, we evaluated the PCNA of β cells by staining for PCNA. The PCNA of 13-week-old OZ-treated mice (2.56 ± 0.48%) was significantly higher than that of control mice (0.92 ± 0.27%) (Fig. 3a,b).

AAT, a naturally-occurring anti-inflammatory glycoprotein, may be beneficial in T1DM by protecting residual β cell function and mass and by exerting anti-inflammatory effects^29^,^30^. We detected α1- antitrypsin (AAT) in serum and in supernatants of DCs in diabetic mice. AAT secreted by DCs from OZ-treated NOD mice as well as AAT levels in serum were elevated compared with PBS-treated DCs, consistent with a protective effects of OZ on β cell function. The results indicated that preserved islet function produced by the TAK1 inhibitor was associated with an elevated production of AAT (Fig. 3c).

### TAK1 inhibits NF-κB and JNK/AP-1 signaling pathways

**Figure 4:**
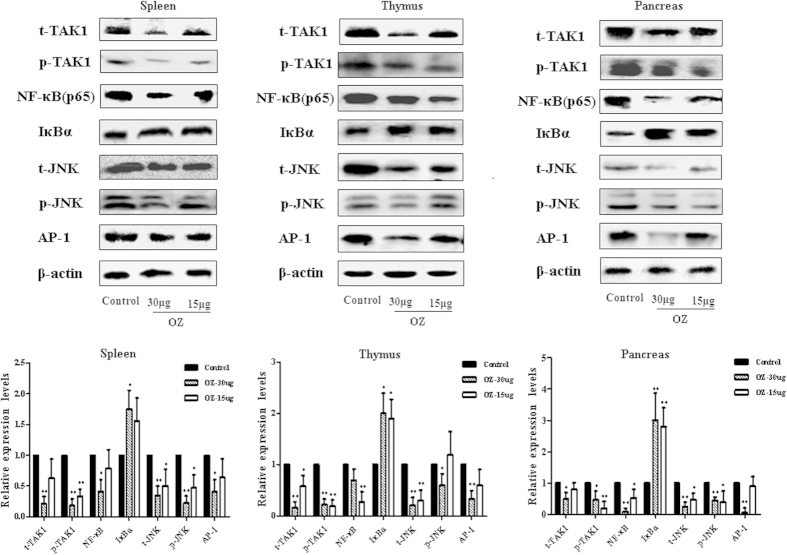
TAK1 regulates the activation of NF-κB and JNK signaling in NOD mice. Thymus, spleen and pancreas from 13-week-old NOD mice treated with OZ for 4 weeks were used to analyze expression of t-TAK1, p-TAK1, NF-κB, IκBα, t-JNK, p-JNK and AP-1 by western blotting, which were all normalized to control. Data are representative of three independent experiments. Data represent the mean ± SD (n = 6 in each group). *P<0.05, **P < 0.01 for Control vs. OZ (15 μg or 30 μg/mouse)

Levels of TAK1 and downstream signaling molecules were quantified by western blotting analysis in spleen, thymus, and pancreatic tissues of PBS- and OZ- treated NOD mice (Fig. 4). OZ treatment significantly interfered with TAK1 expression and TAK1 activation in immune organs and pancreatic tissues compared with PBS-treated mice. The TAK1 inhibitor dramatically inhibited the inflammatory NF-κB signaling cascade, as evidenced by the elevated expression levels of the protease-sensitive inhibitor IκBα. In all immune organs and pancreatic tissues, the TAK1 inhibitor reduced levels of NF-κB which led to accumulation of substantial amounts of total IκBα, reflecting the effective shut-off of inflammatory NF-κB-dependent signals. JNK/AP-1 pathway activation also seemed to be markedly inhibited by the TAK1 inhibitor, as evidenced by reduced total and phosphorylated JNK kinases and AP-1 in comparison with levels in the PBS-treated NOD mice. As AP-1 activation is regulated by phosphorylation of JNK, which belongs to the MAPKs, lower expression of JNK resulted in failure of AP-1 induction.

### TAK1 inhibition elicits a Th1 to Th2 cytokine shift

**Figure 5:**
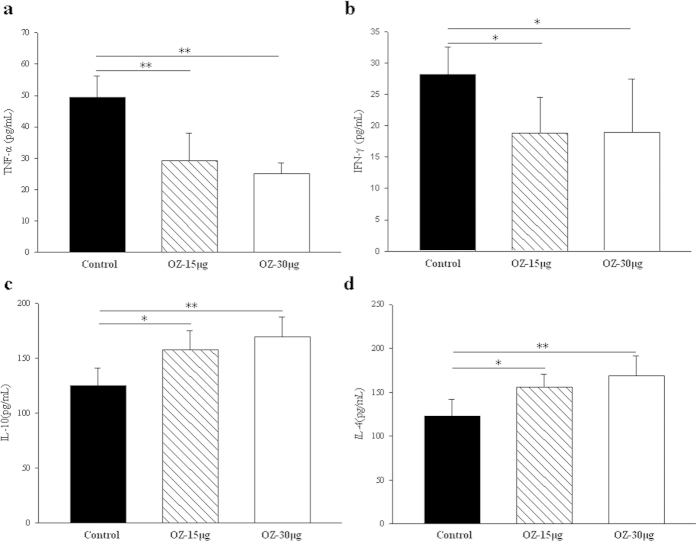
TAK1 inhibitor elicits a Th1 to Th2 cytokine shift. Cells were isolated from spleen of NOD mice at 13 weeks of age previously treated from 8 to 11 weeks of age with PBS, or OZ (15 μg or 30 μg/mouse). Then cells were cultured for 3 days in 96-well culture plates coated with 3 μg/mL anti-CD3/CD28 antibodies. Supernatants were assayed for TNF-α, IFN-γ, IL-4 and IL-10 production by ELISA. Data are representative of three independent experiments. The results of duplicate cultures are expressed as mean ± SD (n = 6 in each group). *P < 0.05, **P < 0.01 for Control vs. OZ (15 μg or 30 μg/mouse)

It has been previously reported that the pathogenic activity of autoreactive diabetogenic T cells in NOD mice can be inhibited if cytokines shift from a Th1 (primarily TNF-α, IFN-γ) to a Th2 (primarily IL-4 and IL-10) profile^31^. To examine whether a Th1 to Th2 cytokine shift happened in NOD mice after injection of TAK1 inhibitor, cells were isolated from spleen of NOD mice at 13 weeks of age previously treated from 8 to 11 weeks of age with PBS or OZ (15 μg or 30 μg/mouse). We analyzed TNF-α, IFN-γ, IL-4 and IL-10 secretion in NOD mice after TAK1 inhibitor treatment, focusing on cultured T lymphocytes supernatants (Fig. 5a–d). OZ treatment significantly reduced Th1 cytokine IFN-γ and TNF-α, and increased Th2 cytokine IL-4 and IL-10, consistent with a Th1 to Th2 cytokine shift in spleen.

### TAK1 inhibition alters DCs and Tregs percentage and characteristics *in vivo*

**Figure 6:**
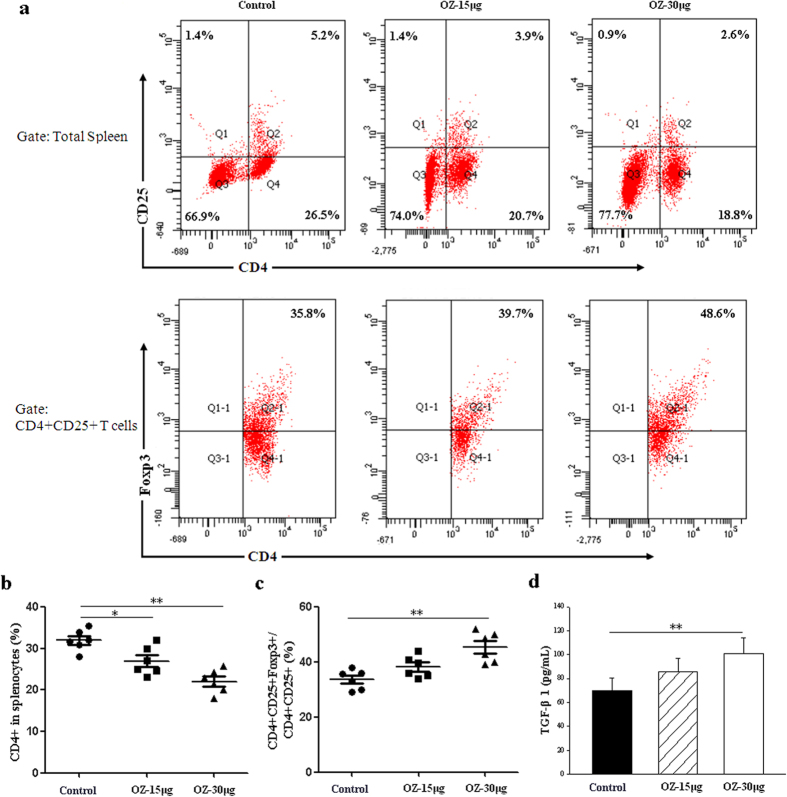
TAK1 regulates Tregs generation and function. T cells were isolated from spleens using standard procedures from 13 week-old NOD mice treated with 15 μg, or 30 μg/mouse OZ once a week from 8 until 11 weeks of age. (**a**) Flow cytometry of T cells population in NOD mice. Cells were stained with CD4, CD25 and Foxp3. (**b**,**c**) The proportion of T cells populations in NOD mice. (**d**) T cells suspensions were prepared and cultured for 3 days in 96-well culture plates coated with 3 μg/mL anti-CD3/CD28 antibodies. Supernatants were assayed for TGF-β1 production in T cells by ELISA. Data are representative of three or four independent experiments. Each symbol represents one mouse. The results of duplicate cultures are expressed as mean ± SD (n = 6 in each group). *P < 0.05, **P < 0.01 for Control vs. OZ (15 μg or 30 μg/mouse)

To establish whether effects of OZ on immunosuppression were related to a modification of Tregs frequency or characteristics, we quantified their proportion in the spleen, thymus and lymph nodes of 13-week-old NOD mice. Administration of OZ for 4 weeks induces major changes in frequencies of CD4^+^ T cells in lymphoid tissues. OZ also increased expression of CD4^+^CD25^+^Foxp3^+^Tregs in a dose dependent manner (Fig. 6a–c and Supplementary Fig. S5–6 online) as well as the production of TGF-β1 in T lymphocytes supernatants as well (Fig. 6d).

**Figure 7:**
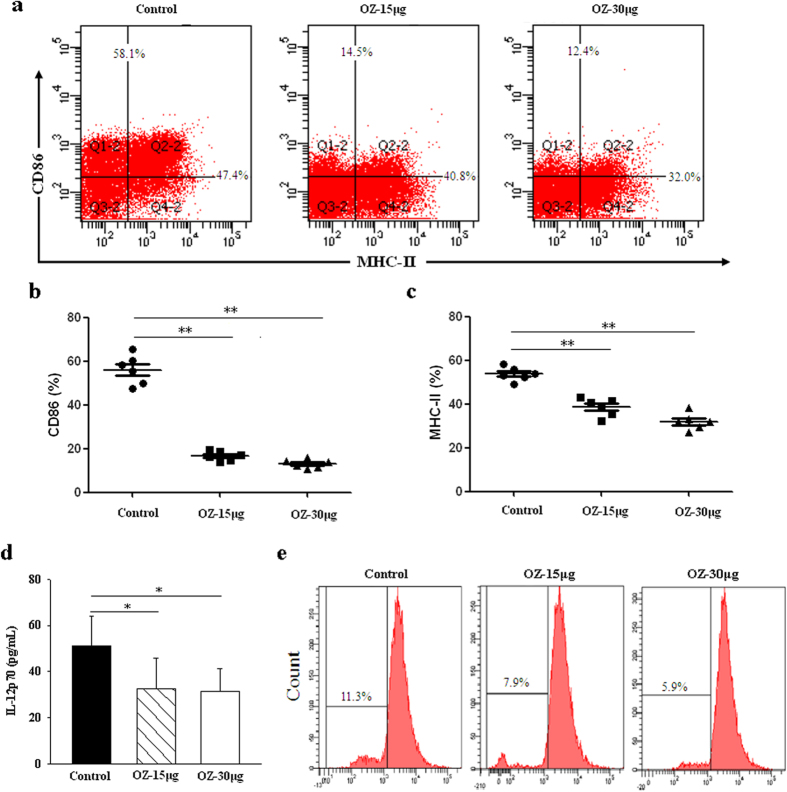
TAK1 regulates DC maturation and function. Bone marrow cells were obtained from 13-week-old NOD mice and cultured in 1 × 10^6^ cells/mL RPMI-1640 containing 10% heat-inactivated FBS in 6-well plates with mouse GM-CSF mouse IL-4. One day 7, cells purified and stained with CD11c, MHC-II and CD86. And supernatants were assayed for IL-12p70 production by ELISA. (**a**) Flow cytometry of DCs populations in NOD mice. (**b**,**c**) The proportion of DCs populations in NOD mice. (**d**) Expression of IL-12p70 in DCs was measured by ELISA. (**e**) The stimulatory capacity of DCs was checked in MLR, OZ-treated DCs impaired T cell proliferation. Allogeneic splenic T cells were stained with diluted carboxyfluorescein succinimidyl ester (CFSE) at 5 × 10^6^ cells/ml for 15 min at 37 °C. DCs from NOD mice as stimulators to stimulate allogeneic splenic T cells from NOD mice in 72 h MLR using 24-well plates. Data are representative of three or four independent experiments. Each symbol represents one mouse. Data represent the mean ± SD (n = 6 in each group). *P < 0.05, **P < 0.01 for Control vs. OZ (15 μg or 30 μg/mouse)

TAK1 inhibition did not affect the differentiation of DC precursors into myeloid DCs *ex vivo*. Myeloid DCs were generated from mouse BM cells (lineage depleted) cultured with GM-CSF plus IL-4. The cell surface expression of CD11 reached about 90% by 7 days in both the control and the TAK1 inhibitor group. However, the TAK1 inhibitor did suppress MHC class II (I-Ak) and CD86, both markers of DC maturity (Fig. 7a–c). DCs from NOD mice treated with either OZ or PBS stimulated T cells as quantified by the mixed leukocyte reaction (MLR). In contrast, OZ-treated DCs impaired T cells proliferation, indicating that the stimulatory capacity of DCs assessed by allogeneic MLR was significantly inhibited (Fig. 7e).

Supernatants of DCs were then used to measure levels of cytokine IL-12p70. As predicted by their low surface levels of MHC-II and costimulatory molecules, IL-12p70 production by DCs isolated from OZ treated mice was also decreased significantly below control levels (Fig. 7d). Collectively, these data provide evidence that TAK1 inhibition alters DCs such that they remain immature for longer periods, and exhibit lower T cell stimulating capability, leading to immunosuppression in T1DM.

### TAK1 inhibition reverses T1DM in cyclophosphamide-accelerated diabetes

**Figure 8:**
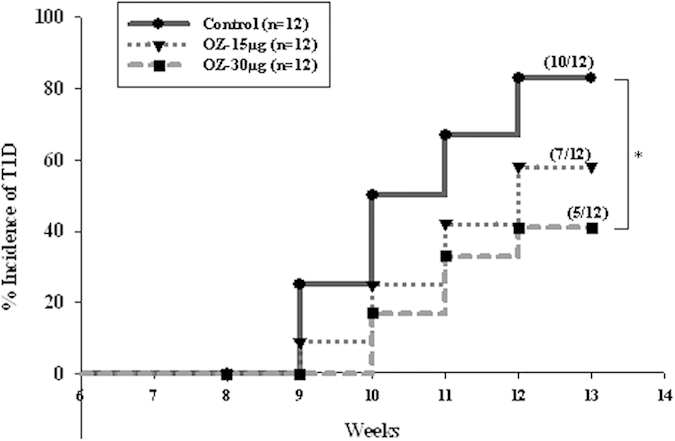
TAK1 inhibitor delays the onset and decreases the incidence of autoimmune diseases in CY-accelerated NOD mice. Accelerated autoimmune diabetes onset was studied in 9-week-old female NOD mice receiving single cyclophosphamide (CY; 200 mg/kg; Sigma-Aldrich) injection intraperitoneally. Experimental groups received OZ at doses of 15 μg or 30 μg/mouse starting 1 week before CY once a week for 4 weeks, respectively. *P < 0.05, compared with PBS-treated NOD mice.

Administration of high-dose cyclophosphamide (CY) to prediabetic NOD mice leads to rapid synchronous onset of T1D^32^,^33^. This is associated with cytotoxicity in a number of lymphoid populations including B cells, and reductions in numbers of CD4^+^CD25^+^Foxp3^+^ regulatory T cells. In keeping with previous study, CY treatment here accelerated diabetes in NOD mice within 14 days. Prior treatment of with the TAK1 inhibitor delayed and reduced the incidence of CY-induced diabetes in a dose dependent manner. A single dose of CY induced diabetes in 83% of untreated mice. Administration of 15 μg or 30 μg/mouse OZ once a week, starting 1 week prior to CY treatment, resulted in a sizable and dose-dependent reduction in the incidence of diabetes. Only 58% and 41% of the animals developed the disease after treatment with the lower and higher OZ doses (Fig. 8).

## Discussion

In this study, TAK1 inhibition with OZ was associated with a significantly reduced incidence of autoimmune diabetes in both spontaneous and accelerated (CY-induced) NOD mouse models. OZ treatment (30 μ g/mouses) decreased the incidence of diabetes by over 40% in both models. Our data also suggest that treatment with the TAK1 inhibitor induced long-term tolerance, since reduced inflammation and islet protection were found after treatment remission for four weeks. The reversal did not require continuous TAK1 blockade, which may have been detrimental for the immune system and even for β-cell function. The protective effect of TAK1 inhibition was not unexpected, because TAK1 is indispensable for cellular responses to Toll-like receptor ligands, CD40, and B cell receptor crosslinking, all potentially implicated in immune-related pathological processes including T1D^15^,^16^.

TAK1 is considered to be a critical regulator of stress responses, immunity, and inflammation; all of these actions are mainly mediated by the downstream pathways p38 MAPK, JNK, extracellular signal–regulated kinases (ERK)-1 and ERK2, and NF-κB p65^26^. NF-κB is sequestered in the cytoplasm as an inactive complex with inhibitors of NF-κB (IκB). With the phosphorylation and degradation of IκB, the p65 subunit of NF-κB translocates into the nucleus where it activates transcription of TNF-α and IL-1β genes^34^,^35^. The JNK/AP-1 signaling pathway acts as a multifunctional regulator of cell survival as it functions as a stress-related inducer of programmed cell death in many tissues^36^,^37^. Recently, it has been confirmed that the role of NF-κB and JNK/AP-1 pathways are implicated in the pathogenesis of T1D. Induction of NF-κB is pro-apoptotic in pancreatic β cells and JNK activation in pancreatic β cells leads to glucose intolerance^38^,^39^. Thus, in line with previous study, our results here indicates that TAK1 inhibition blocks JNK and NF-κB activation in immune organs and pancreatic tissues, effectively shutting off inflammatory NF-κB - and JNK - dependent pathways.

Enhanced antigen presentation cell (APC) function due to elevated NF-κB activation correlates with the progression of autoimmune diabetes in NOD mice^40^. Here, we report that inhibition of TAK1 down-regulated CD86 and MHC-II expressions in DCs, resulting in low levels of the proinflammatory cytokine IL-12p70. *In vitro*, blockade of TAK1 inactivated NF-κB and JNK/AP-1 signaling pathways, leading to apoptotic death of mature DCs. As professional antigen-presenting cells, DCs are important for the induction of both adaptive immunity and tolerance^41^. Tolerogenic DCs exhibiting low expressions of CD80, CD86, CD40, MHC-II are beneficial for T1DM^42^. Elevated NF-κB activation results in an overall enhanced APC function of DCs in NOD mice^40^. Thus, the ability of TAK1 inhibition to down-regulate TAK1-dependent pathways may help to explain the observed reductions in diabetes incidence and preserved islet function in OZ-treated NOD mice. These results are consistent with those of previous reports showing that deficiency of TAK1 in dendritic cells from lymphoid and nonlymphoid tissues affects immune homeostasis by disrupting T cell homeostasis and preventing effective T cell priming and generation of regulatory T cells^25^.

Treg cells may exert beneficial effects during the development of T1D^28^. Treg cell maintenance inhibits the progression of autoimmune responses at least in part by interfering with the function of DCs, the only major APC subset involved in activating T lymphocytes responses to self-antigen^43^. In the NOD mouse, DC-expanded, islet-specific Tregs conferred protection and restored normoglycemia in overt diabetes^44^. Although a previous study indicated that TAK1 in DCs mediated the induction of antigen-specific iTreg cells both *in vivo* and *in vitro*^25^, we report here an increase in Foxp3^+^ Tregs was associated with elevated levels of TGF-β1 in NOD mice after OZ treatment. Treg cells also inhibit the expression of CD86 and CD80 costimulatory molecules on DC by down-regulating the activation of NF-κB, and this effect also requires TGF-β1^45^. TGF-β1 negatively modulates DC maturation and favors the differentiation of tolerogenic DC^46^. Therefore, Treg cells appear to act on immature DCs to block the up-regulation of costimulatory molecules such as CD80 and CD86^47^. In feedback fashion, tolerogenic DCs, through enhanced production of IDO, subsequently promote Treg cell differentiation and expansion^46^. TGF-β1 is a stimulator of TAK1 signaling, which would be expected to exacerbate disease in NOD mice. This did not occur in this study, perhaps because immunological tolerance had been established after short-term OZ treatment. To investigate this possibility further, we measured changes of TGF-β1 during OZ treatment and found that levels of TGF-β1 decreased during the first three weeks after treatment, but began to increase beginning after the fourth week (data not shown). These results indicated that increased TGF-β1 levels contributed to the establishment of immune tolerance subsequent to OZ treatment. Thus, TAK1 inhibition by OZ treatment may have been protective against diabetes in NOD mice in part by promoting immunosuppressive interactions between Tregs and DCs.

Importantly, TAK1 inhibition with OZ inhibited systemic and islet inflammation, as evidenced by decreased lymphocytic infiltration and proinflammatory cytokine production. Systemic TAK1 blockade led to significantly impaired production of Th1-type cytokines (TNF-α, IFN-γ) and increased Th2-type cytokine (IL-4 and IL-10) secretion from T cells in NOD mice. These findings may represent a novel role of TAK1 in immune-mediated inflammatory disorders.

Apart from balancing immune responses to reverse diabetes, blockade of TAK1 also preserved β cell function. We found that the reversal of diabetes after treatment with OZ was primarily due to decreased β cell death rather than inhibited growth of new cells. When administered to NOD mice at age 15 weeks, OZ did not reverse diabetes (data not shown). This suggested that residual β cells need to be present for OZ to be effective. When administered to 8-week-old prediabetic NOD mice, OZ delayed the onset and reduced the incidence of autoimmune diabetes. This protective effects was associated with a 48.1% decrease in apoptotic β cells. OZ treatment of younger NOD mice also reduced insulitis in islets and preserved insulin-positive islets. OZ treatment also increased levels of AAT both in blood and in supernatants of DCs. AAT, an acute-phase reactant with serine proteinase inhibitor, possesses anti-inflammatory and anti-apoptotic effects^48^.AAT also exerts cytoprotective effects upon islets *in vitro* and preserves residual β cell function in NOD mice^29^,^30^. Thus, inhibition of TAK-1 seems to decrease the severity and incidence of diabetes in NOD mice at least in part by promoting the levels and actions of the antiinflammatory cytokine AAT and consequently enhancing the survival of β cells.

It has been reported that TAK1 negatively regulated NF-κB and p38 MAPK activation in Gr-1^+^CD11b^+^ neutrophils^26^, In contrast, we demonstrated here that TAK1 was a key regulator of T1DM whose inhibition delayed and reduced the incidence of diabetes by alleviating insulitis and preserving islet function. Inhibition of TAK1 also induced Tregs and immature DCs in secondary lymphoid organs. The net effect of TAK1 inhibition in NOD mice thus appears to be protective rather than disease-enhancing. Strategies specifically targeting TAK1 might therefore prove to be useful for the treatment of autoimmune diabetes without suppressing immune responses peripherally.

## Materials and Methods

### Mice

Animal experiments were approved by *the Institutional Animals Care and Use Committee of Tongji Medical College, Huazhong University of Science and Technology*. Animal care and experimental procedures were carried out in accordance with *the guidelines of the Institutional Animal Care and Use Committee of Tongji Medical College and the National Institutes of Health Guide for the Care and Use of Laboratory Animals.* Female NOD/ShiLtJ mice and C57BL/6 mice were purchased from Beijing HFK Bio-Technology Co. Ltd. and kept in a specific pathogen-free environment.

### TAK1 inhibitor (OZ) administration in NOD mice

Spontaneous autoimmune diabetes was evaluated in female NOD mice exposed to either 15 μg/mouse/week OZ, 30 μg/mouse/week OZ, or PBS treatment starting at age 8 weeks up to age 11 weeks, each group had 12 mice.

At the end of the 4-week treatment period, 6 mice per group (13-weeks old) were randomly selected for determining cytokine levels, histopathological insulitis, DCs surface molecules and immunostimulatory function, the percentage of CD4^+^CD25^+^Foxp3^+^ Tregs, and TAK1 expression. The remaining 6 mice per group were fed until 40 weeks of age for diabetes incidence studies. Nonfasting blood glucose was measured every week with an Easy Check monitor (Home Aide Diagnostics, Deerfield Beach, FL) beginning at age of 10 weeks. Mice were considered diabetic when blood glucose concentrations exceeded 250 mg/dL on two consecutive determinations.

### Intraperitoneal glucose tolerance test (IPGTT)

IPGTT analyses were performed in age-matched NOD mice including spontaneous NOD mice and OZ-treated spontaneous NOD mice at 12 weeks of age. Food was withheld 16 h before testing. Animals were weighed and blood glucose concentrations were measured just before injection with 2 g/kg of glucose (i.p.). Glucose concentrations were measured at 30, 60, 90, 120, 180 min after glucose injection.

### Insulitis score and immunohistochemistry analysis

Pancreata from NOD mice treated with OZ or PBS for 4 weeks were removed at age 13 weeks. They were fixed in 10% formalin, embedded in paraffin, and stained with hematoxylin and eosin (H&E). Insulitis score was assigned under double-blinded conditions and the degree of insulitis was determined (0 = no insulitis; 1 = periinsulitis; 2 = invasive insulitis with <50% islet area affected; 3 = invasive insulitis with >50% islet area affected). Average percentages of insulitis were determined from 50 to 100 islets in each treatment group.

Other sections were stained for insulin and TAK1 using anti-insulin and anti-TAK1 antibody, visualized with diaminobenzidine. Apoptotic and proliferated cells in pancreatic islets were determined by terminal deoxynucleotidyl transferase-mediated dUTP nick-end labeling (TUNEL) and proliferating cell nuclear antigen (PCNA) staining. The level of apoptotic or proliferated cells was expressed as the number of TUNEL or PCNA-positive cells per area of pancreatic islets (cells/mm^2^).

### Generation of DCs from bone marrow

Bone marrow cells were obtained from 13-week-old NOD mice which were treated with OZ or PBS for 4 weeks and cultured in (1 × 10^6^ cells/mL) in RPMI-1640 medium containing 10% heat-inactivated fetal bovine serum (FBS, Gibco, USA), 100 U/ml penicillin and 100 ug/ml streptomycin (Gen-view Scientific Inc., USA) in 6-well plates with 20 ng/mL mouse granulocyte macrophage-colony stimulating factor (mGM-CSF, Signalway Antibody LLC, USA), and 10 ng/mL mouse interleukin (IL)-4 (Signalway Antibody LLC, USA). On day 7, the cells were washed thoroughly and CD11c^+^DC was purified to >90% using anti-CD11c immunomagnetic beads (Miltenyi Biotec, Bergisch Gladbach, NRW). Supernatants were assayed for AAT and IL-12p70 production by ELISA.

### Generation of T cells

T cells were isolated from the spleen, thymus and lymph nodes of control, OZ-treated NOD mice by anti-CD4 immunomagnetic beads (Miltenyi Biotec, Bergisch Gladbach, NRW). T cells (2 × 10^5^) were cultured for 3 days in 96-well culture plates coated with 3 μg/mL anti-CD3/CD28 antibodies. Transforming growth factor (TGF)-β1, interferon (IFN)-γ, IL-4, IL-10 and tumor necrosis factor (TNF)-α production were assayed in culture supernatants using ELISA.

### Mixed leukocyte reaction (MLR)

DCs from NOD mice as stimulators were treated with 0.5 mg/mL mitomycin C to prevent proliferation, and then stimulated with allogeneic T cells labeled for 20 min with 5-carboxyfluorescein diacetate succinimidyl ester (CFSE; 2 μM; Sigma, USA) from NOD mice in 72 h MLR using 24 well rounded bottom plates as described previously.

### Protein analysis

Cells or tissues were lysed in Radio-Immunoprecipitation Assay (RIPA) buffer. Lysates were separated by SDS-polyacrylamide gels (SDS-PAGE) and were transferred onto polyvinylidene difluoride (PVDF) membranes (BioRad). After membranes were incubated with primary antibodies overnight at 4 °C, appropriate horseradish peroxidase (HRP)-conjugated secondary antibodies were applied for 1–2 h at room temperature, prior to detection with an enhanced chemiluminescence system (Perkin-Elmer). Polyclonal antibody to anti-TLR4, anti-TAK1, anti-p-TAK1 (Ser 192), anti-JNK, anti-p-JNK (Thr 183/Tyr 185), anti-NF-κB p65, anti-IκBα, anti-AP-1 were purchased from Santa Cruz. Biotechnology. Densitometric analyses of protein abundance were determined by Image J software and the amount of protein expression was normalized against β-actin added to the same sample.

### Flow cytometry analysis

Cells suspensions were prepared from spleen, thymus or lymph nodes of NOD mice, previously treated with OZ or PBS for 4 weeks, using standard procedures. DC single-cell suspensions were prepared from bone marrow following standard procedures. Single-cell suspensions were stained with antibodies against the following cell surface antigens: fluorescein isothiocyanate (FITC)-conjugated anti-CD86, allophycocyanin (APC)-conjugated anti-CD11c and phycoerythrin (PE)-conjugated anti-MHC-II (eBioscience, USA); w/PE-Cy5 Foxp3, FITC-CD4, PE-CD25 (eBioscience, USA). Analysis was performed on a BD LSRII flow cytometer with FACSD via software (BD Pharmingen). Postacquisition analysis was performed with Flowjo software version 9.1.

### Cytokine quantification

Supernatants from spleen T cell or DC cultures and serum from OZ-treated NOD mice or control mice were stored until quantification of mouse IL-4, IL-10, IL-12p70, TGF-β1, IFN-γ, TNF-α, α1-antitrypsin (AAT) using specific quantitative enzyme-linked immunosorbent assay (ELISA) kits (eBioscience, USA).

### Accelerated experiment

Nine-week-old female NOD mice were randomly divided into 3 groups of 12 animals in each group. Accelerated autoimmune diabetes onset was studied in NOD mice receiving a single cyclophosphamide (CY; 200 mg/kg; Sigma-Aldrich) injection intraperitoneally (i.p.). Experimental groups received TAK1 inhibitor -OZ- starting 1 week before CY. OZ was administered to NOD mice at doses of 15 or 30 μg/mouse once a week for 4 weeks. The development of diabetes was monitored according to above description.

### *In vitro* assays

To study the effect of OZ on DCs *in vitro*, DCs were generated from bone marrow cells of C57BL/6 mice as above. DCs were treated with GM-CSF and IL-4 from the first day. On day 7, cells were stimulated with fresh medium containing lipopolysaccharide (LPS; Sigma-Aldrich; 1 μg/mL) for 24 h, then cells were treated with either DMSO vehicle or 5 μM OZ for 4 h before subsequent experiments. FITC annexin V/PI staining was used to detect the stimulation-induced apoptosis in OZ-treated DCs by FACS analysis. To detect effects of OZ on maturity and survival of DCs, single-cell suspensions were stained with antibodies against the following cell surface antigens: FITC-conjugated anti-CD86, APC-conjugated anti-CD11c and PE-conjugated anti-MHC-II (eBioscience, USA). Western blotting was used to analyze TLR4-induced signal transduction in DCs. The cells were treated with 1 μg/ml LPS for 24 h.

To evaluate the effect of OZ on Tregs generation *in vitro*, T cells from spleen, thymus and lymph nodes of C57BL/6 mice were isolated by anti-CD4 MACS. Then T cells (5 × 10^5^) were cultured for 72 h in 6-well culture plates supplementary with 3 μg/mL anti-CD3/CD28 antibodies, and cells were treated with either DMSO vehicle or TAK1 inhibitor (OZ, 5 μM) for 4 h before subsequent experiments. Mouse Regulatory T cell Staining Kit (w/PE-Cy5 Foxp3 FJK-16s, FITC CD4, PE CD25; Treg Kit) was used to detect regulatory T cells according to the manufacturer’s instructions, and then analyzed for the percentage of CD4^+^CD25^+^Foxp3^+^ Tregs by flow cytometry. Foxp3 mRNA level of T cells were determined by qRT-PCR.

### Cytotoxicity test

T cells and bone marrow-derived DCs were obtained from C57BL/6 mice as above description. Then DCs or T cells were incubated with 0–100 μM OZ for 12 h and its cytotoxicity was measured using a Cell Counting Kit-8 (Dojindo Inc., Rockville, MD).

### RNA analysis *in vitro*

Total RNA was isolated from prepared T cells and DCs using MagZol reagent (Invitrogen, Carlsbad, CA, USA) according to the manufacturer’s protocol. Quantitative real-time PCR was carried out in an ABI 7900 real-time PCR system, with all target gene primers purchased commercially (Applied Biosystems). Cycling conditions were as follows: polymerase activation at 95 °C for 10 min, 40 cycles of denaturation at 95 °C for 30 s, annealing at 60 °C for 20 s and extension at 72 °C for 30 s. Results were analyzed using SDS 2.4 software (Applied Biosystems). mRNA levels of target genes were normalized to those of β-actin. All experiments were repeated 4 times per specimen with consistent results.

### Statistical analysis

Data were expressed as the mean ± standard deviation (S.D.). Each data point presented in scatter plots was from a single mouse. Statistical analysis was performed using SPSS software, Version 13.0. Survival curves were compared by Kaplan Meier log-rank test. One Way ANOVA followed by Bonferroni’s post-hoc test (for parametric data) or Kruskal-Wallis test with Dunn’s Multiple Comparison post-test (for nonparametric data) were used to evaluate differences between groups. Results were considered statistically significant if *P *< 0.05.

## Additional Information

**How to cite this article**: Cao, H. *et al.* TAK1 inhibition prevents the development of autoimmune diabetes in NOD mice. *Sci. Rep.* **5**, 14593; doi: 10.1038/srep14593 (2015).

## Supplementary Material

Supplementary Information
