# Supplementary material for: TAK1 inhibition prevents the development of autoimmune diabetes in NOD mice
Source: Sci Rep. 2015 Oct 13;5:14593. doi: 10.1038/srep14593 (PMC4602205; doi:10.1038/srep14593)
Supplement: Supplementary Information [file srep14593-s1.pdf]

TAK1 inhibition prevents the development of autoimmune diabetes in NOD mice

Hui Cao[1#], Jingli Lu[1#], Jiao Du[1], Fei Xia[1], Shouguo Wei[1], Xiulan Liu[1], Tingting Liu[1], Yang Liu[2] & Ming Xiang[1]*

1. Department of Pharmacology, School of Pharmacy, Tongji Medical College, Huazhong University of Science and Technology, Wuhan, China

2. Synergy Innovation Center of Biological Peptide Antidiabetics of Hubei Province, School of Life Science, Wuchang University of Technology, Wuhan, China

[#] These authors contributed equally to this work.

***Corresponding author**:

Prof. Ming Xiang

Huazhong University of Science and Technology, Tongji Medical College, School of Pharmacy, Dept. of Pharmacology, NO. 13, Hang Kong Road, 430030 Wuhan, China.

Phone: +862783657813

Fax: +862783657547

E-mail: tjphxm@aliyun.com

Supplementary Figure 1

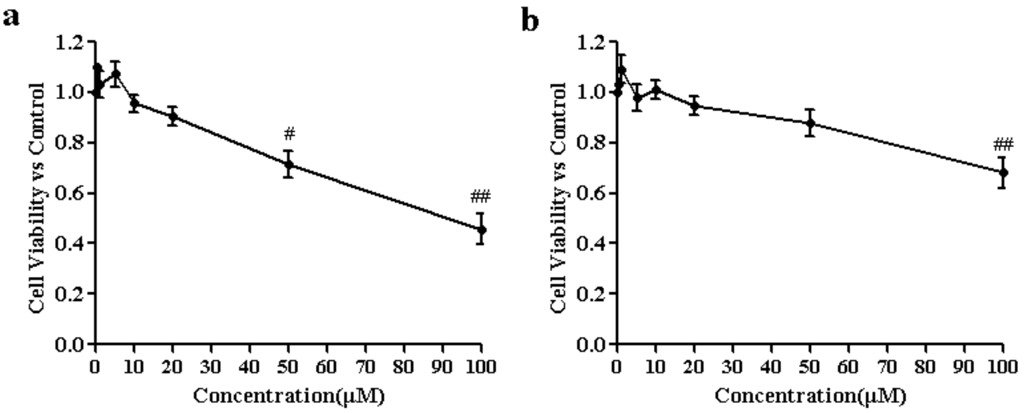

Figure S1 | Cytotoxicity measurement of TAK1 inhibitor (OZ) on DCs and T cells. DCs (a) or T cells (b) were treated with 0-100μM OZ for 12 h for determining cytotoxicity. Data are shown as mean ± SD. $^{\#}P<0.05$, $^{\#\#}P<0.01$ for Control vs. OZ

Supplementary Figure 2

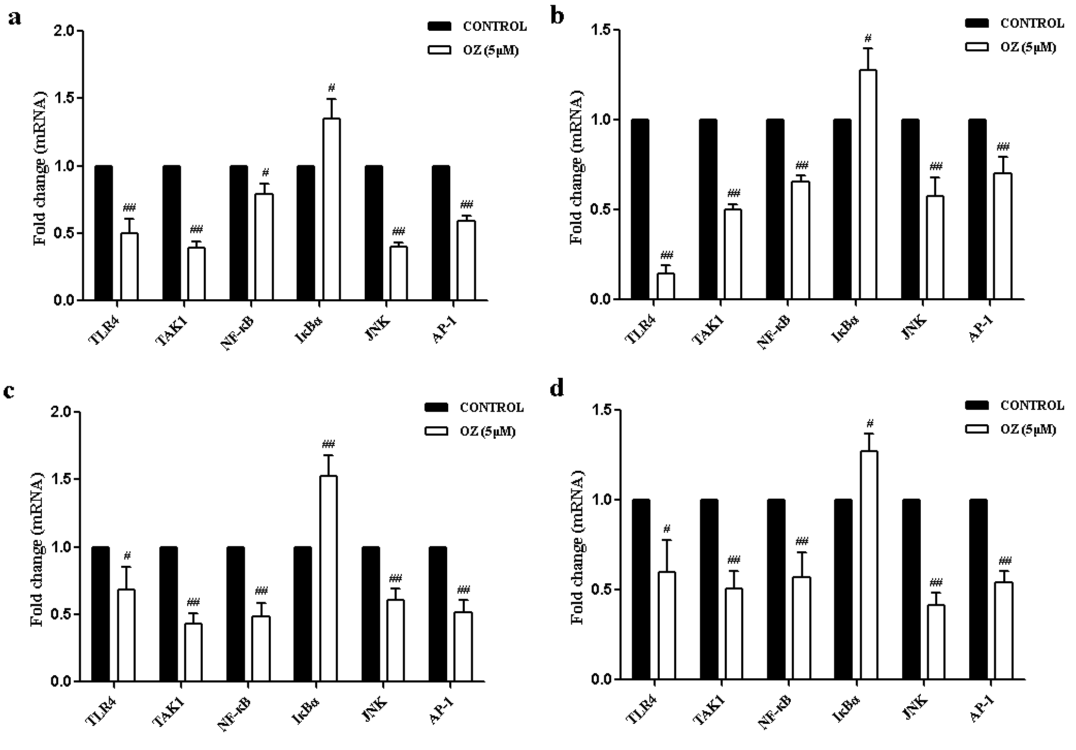

Figure S2 | TAK1 inhibitor (OZ) downregulated the mRNA expression of TLR4, TAK1 and downstream of TAK1 signaling *in vitro*. Related genes expression in bone-marrow derived DCs and T cells from spleen, thymus and lymph nodes were studied using qRT-PCR. (a) DCs. (b) T cells from spleen. (c) T cells from thymus. (d) T cells from lymph nodes. The key genes downstream of TAK1 signaling, including NF-κB, IκBα, JNK, AP-1. Values are presented as mean ± SD, $^{\#}P < 0.05$, $^{\#\#}P < 0.01$ for Control vs. OZ (5μM)

Supplementary Figure 3

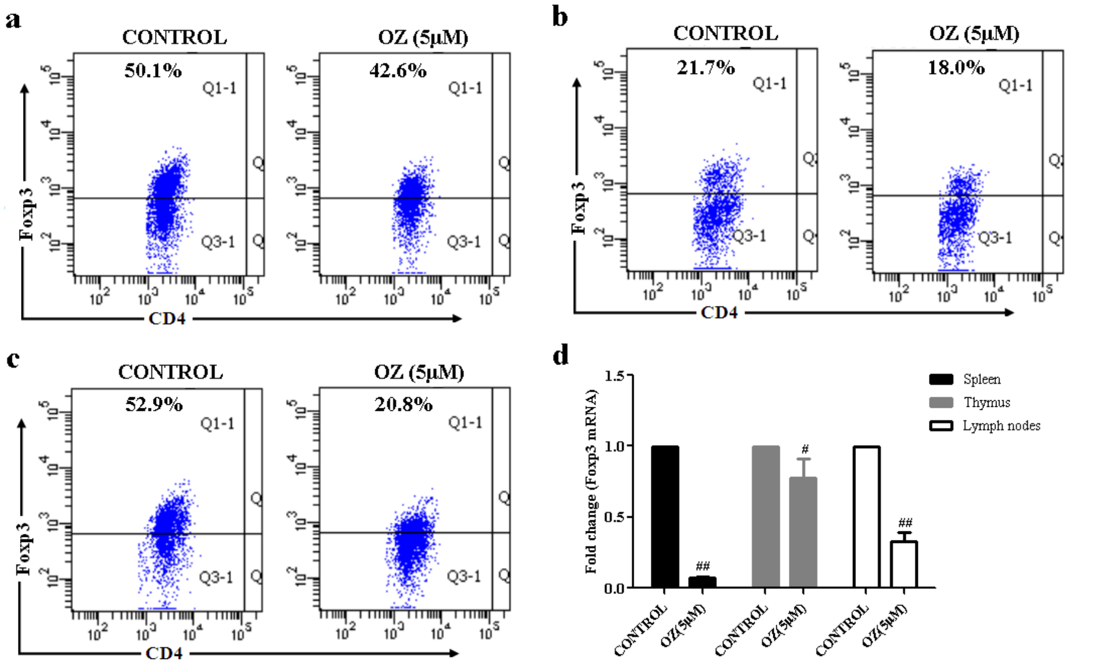

Figure S3 | Effect of TAK1 inhibitor (OZ) on Tregs generation and Foxp3 gene expression *in vitro*. Representative FACS staining for Foxp3 on gated $CD4^{+}$ $CD25^{+}$T cells in spleen (a), thymus (b) and lymph nodes (c) isolated from C57BL/6 mice. (d) Foxp3 mRNA expression in spleen, thymus and lymph nodes was detected by qRT-PCR. Results are expressed as mean ±SD. $^{\#}P<0.05$, $^{\#\#}P<0.01$ for Control vs. OZ (5μM)

Supplementary Figure 4

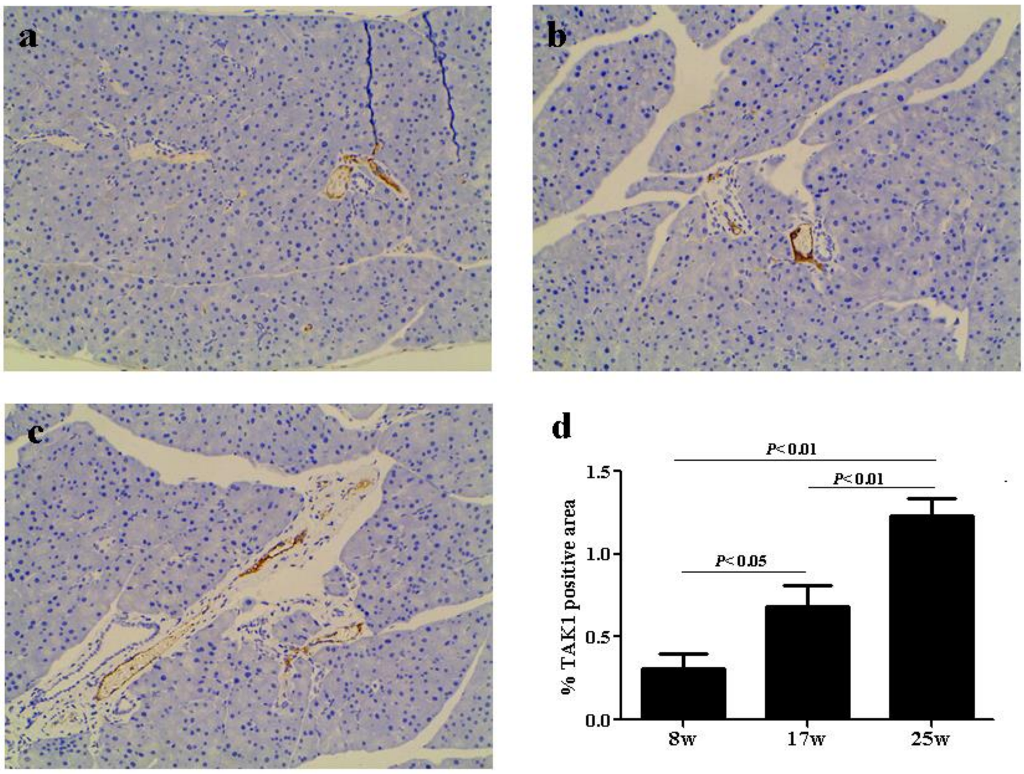

Figure S4 | TAK1 distributed in the pancreatic exocrine compartment. Pancreata were removed from NOD mice of different weeks of age and were immunostained with anti-TAK1 antibody. (a) 8-week-old NOD mice. (b) 17-week-old NOD mice. (c) 25-week-old NOD mice. (d) quantification of TAK1-positive area. Results are shown as mean ±SD.

Supplementary Figure 5

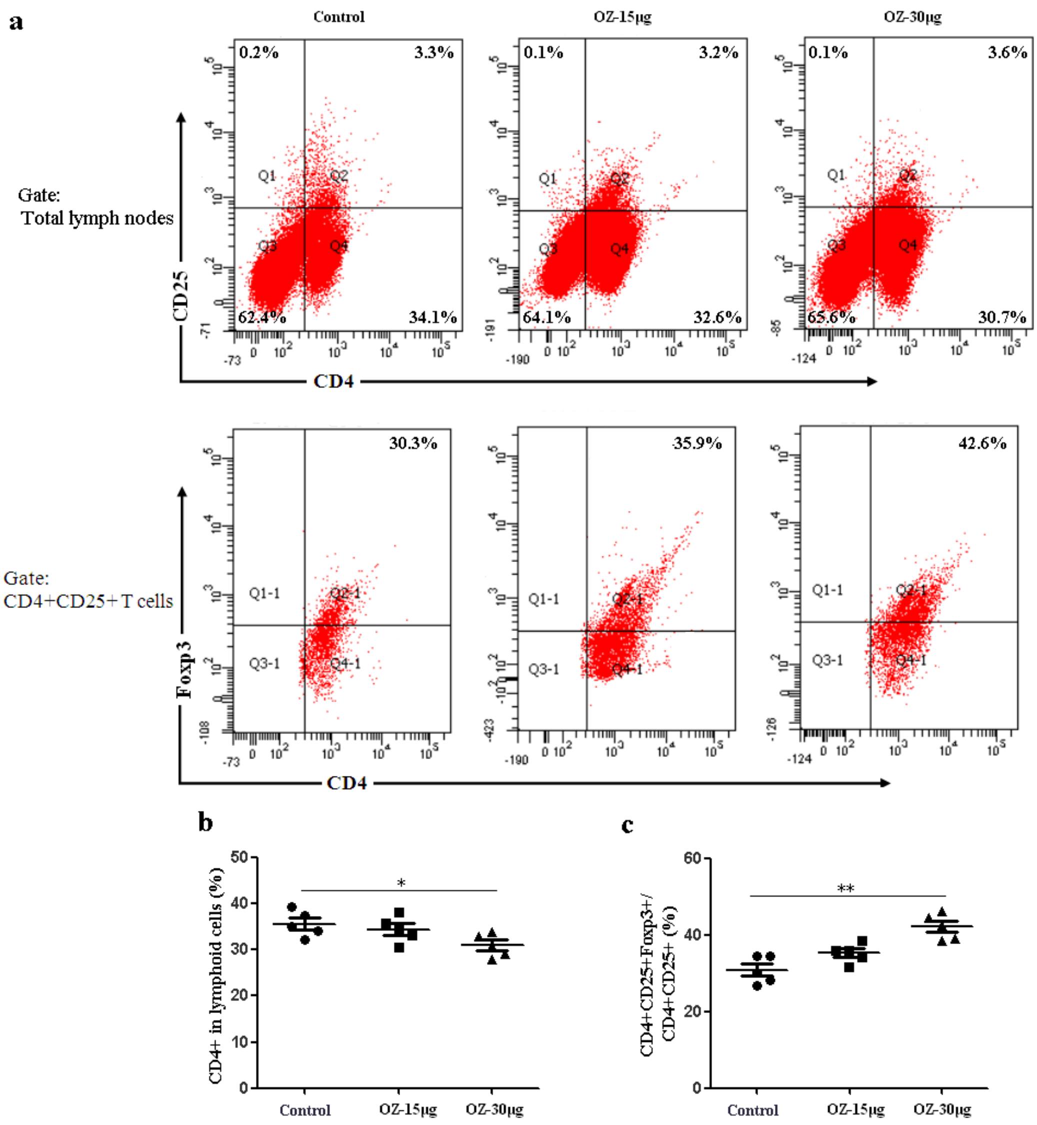

Figure S5 | Effects of TAK1 inhibition on CD4$^+$CD25$^+$Foxp3$^+$ **Tregs differentiation.** T cells were isolated from lymph nodes using standard procedures from 13 week-old NOD mice treated with 15μg, or 30μg/mouse once a week from 8 until 11 weeks of age. (a) Representative FACS staining for Foxp3 on gated CD4$^+$CD25$^+$T cells. (b, c) The proportion of CD4$^+$T cells and CD4$^+$CD25$^+$Foxp3$^+$ Tregs in lymph nodes from NOD mice. *P<0.05, **P<0.01 for Control vs. OZ (15μg or 30μg/mouse)

Supplementary Figure 6

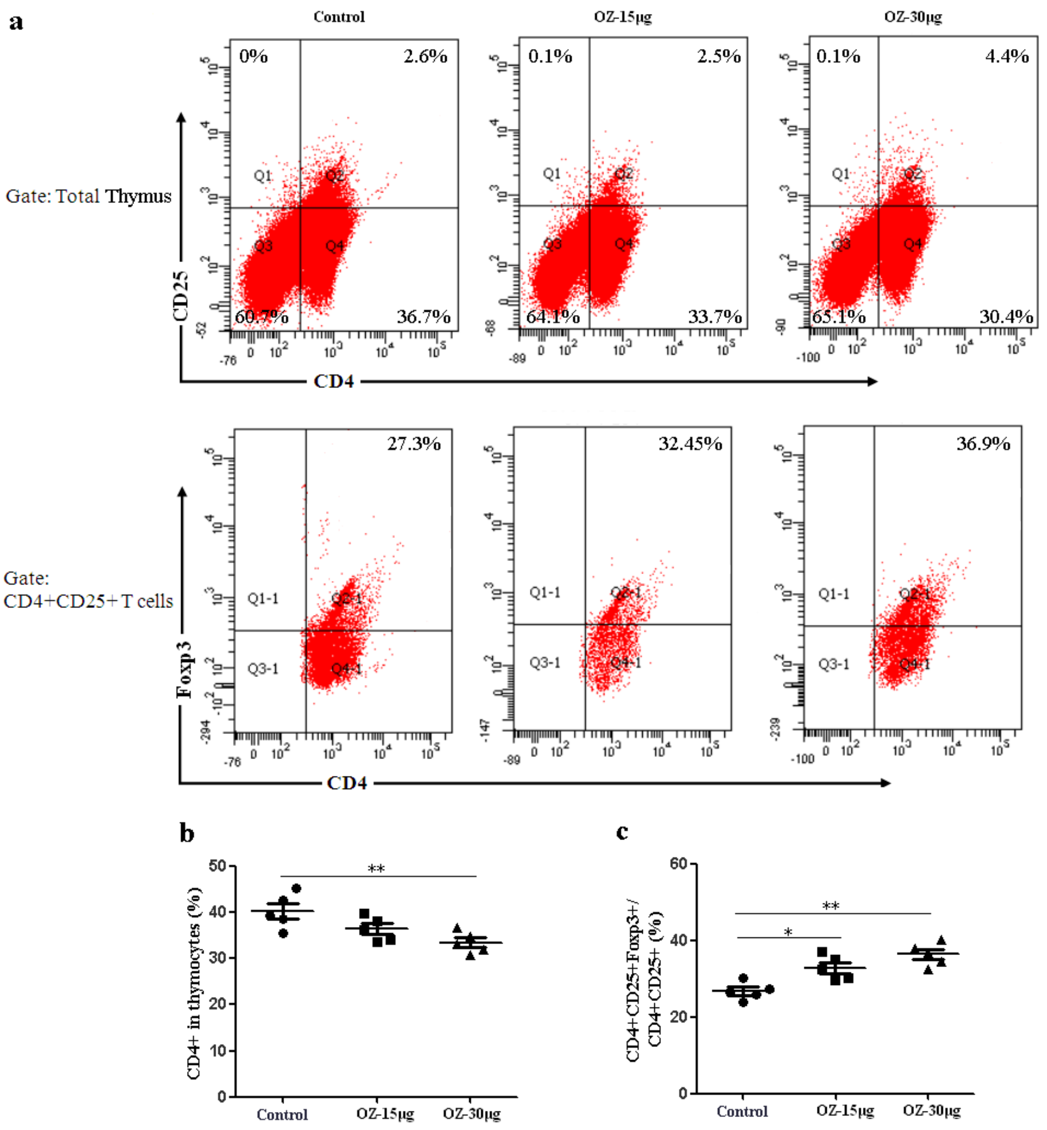

Figure S6 | Effects of TAK1 inhibition on CD4$^+$CD25$^+$Foxp3$^+$ Tregs differentiation. T cells were isolated from thymus using standard procedures from 13 week-old NOD mice treated with 15µg, or 30µg/mouse once a week from 8 until 11 weeks of age. (a) Representative FACS staining for Foxp3 on gated CD4$^+$CD25$^+$T cells. (b, c) The proportion of CD4$^+$T cells and CD4$^+$CD25$^+$Foxp3$^+$ Tregs in thymus from NOD mice. *P<0.05, **P<0.01 for Control vs. OZ (15µg or 30µg/mouse)
